# Effect of Etching Protocols on the Bonding Performance of a HEMA-Free Two-Step Universal Adhesive to Caries-Affected Dentin

**DOI:** 10.3290/j.jad.c_2785

**Published:** 2026-08-18

**Authors:** Melike Bilce, Fatih Bedir, Günes Bulut Eyüboglu, Tugba Serin Kalay, Muhammet Karadas

**Affiliations:** a Melike Bilce Assistant Professor, Department of Restorative Dentistry, Recep Tayyip Erdoğan University, Faculty of Dentistry, Rize, Turkey. Investigation, methodology, and read and approved the final version of the manuscript.; b Fatih Bedir Assistant Professor, Department of Restorative Dentistry, Recep Tayyip Erdoğan University, Faculty of Dentistry, Rize, Turkey. Investigation, methodology, writing (original draft), and read and approved the final version of the manuscript.; c Günes Bulut Eyüboglu Professor, Department of Restorative Dentistry, Karadeniz Technical University, Faculty of Dentistry, Trabzon, Turkey. Proofread the manuscript, performed a certain test, consulted on and performed statistical evaluation, and read and approved the final version of the manuscript.; d Tugba Serin Kalay Professor, Department of Restorative Dentistry, Karadeniz Technical University, Faculty of Dentistry, Trabzon, Turkey. Performed the experiments in partial fulfillment of requirements for a degree, proofread the manuscript, performed a certain test, consulted on and performed statistical evaluation, and read and approved the final version of the manuscript.; e Muhammet Karadas Professor, Department of Restorative Dentistry, Recep Tayyip Erdoğan University, Faculty of Dentistry, Rize, Turkey. Idea, hypothesis, experimental design, performed the experiments in partial fulfillment of requirements for a degree, wrote the manuscript, performed a certain test, consulted on and performed statistical evaluation, and read and approved the final version of the manuscript.

**Keywords:** caries-affected dentin, bond strength, HEMA, universal adhesive

## Abstract

**Purpose:**

To assess the bonding performance of a two-step universal adhesive applied in etch-and-rinse and self-etch protocols to caries-affected dentin.

**Methods and Materials:**

Mid-coronal dentin surfaces were obtained from extracted human molars and assigned to either sound or caries-affected dentin groups. Specimens were randomly distributed into 12 subgroups (n = 10) based on adhesive type (G2-Bond Universal, Gluma Bond Universal, Clearfil S3 Bond Universal), bonding protocol (self-etch or etch-and-rinse), and evaluation period (24 h or aging). Resin composite blocks were fabricated and subjected to shear bond strength testing after 24 h or 10,000 thermocycles. Fourier-transform infrared spectroscopy was employed to measure the degree of conversion of each adhesive. Statistical analyses included ANOVA, Weibull analysis, and Kruskal–Wallis tests (α = 0.05).

**Results:**

Adhesive type and bonding protocol significantly influenced bond strength (*p* < 0.001), whereas substrate condition and thermocycling had no significant impact (*p* > 0.05). After aging, Clearfil S3 Bond Universal in the self-etch protocol presented considerably lower bond strength to caries-affected dentin compared with G2-Bond Universal and Gluma Bond Universal, with no significant difference between the latter two. After aging, Gluma Bond Universal and G2-Bond Universal revealed significantly greater bond strength to caries-affected dentin when applied in the self-etch protocol than in the etch-and-rinse protocol. No significant differences were detected among adhesives when the etch-and-rinse protocol was used, regardless of testing time and substrate.

**Conclusions:**

Phosphoric acid pre-etching negatively affected the bonding performance of Gluma Bond Universal and G2-Bond Universal to caries-affected dentin.

A primary objective of restorative dentistry is the development of adhesive systems that form strong and reliable bonds between the restorative materials and tooth hard structures, thereby preserving mechanical integrity and healthy tissue.^3^ Among recent advancements, universal adhesives (UAs) offer significant versatility because they can be applied using self-etch (S-E), selective-etch, or etch-and-rinse (E-R) strategies.^19,64^ The E-R strategy includes the use of phosphoric acid to remove the smear layer, creating deep microretentive pits in enamel and fully demineralizing dentin to a depth of several micrometers. The formation of micro- and macrotags in enamel, together with hybridized collagen and resin tags in dentin, constitutes the primary mechanism of adhesion.^34,56^ In contrast, the S-E strategy combines etching and priming in a single step using acidic functional monomers, resulting in partial demineralization of dentin.^56^ While the E-R strategy generally provides higher enamel bond strength, the S-E approach may offer advantages in dentin through a combination of micromechanical interlocking and chemical interaction with residual hydroxyapatite.^5,24^ These differences may become clinically relevant when bonding to structurally altered dentin substrates.

Dental adhesives are complex, solvated, heterogeneous blends of hydrophilic and hydrophobic monomers, which may also contain filler particles in suspension. Because dentin is an intrinsically moist and highly hydrophilic substrate, adhesive formulations must exhibit sufficient hydrophilicity to adequately wet the dentin surface and infiltrate the demineralized collagen matrix. 2-hydroxyethyl methacrylate (HEMA) has been extensively incorporated into dental adhesives due to its low molecular weight and hydrophilic properties, facilitating surface wetting in the S-E protocol and enhancing penetration into the demineralized collagen network in the E-R protocol. Moreover, HEMA functions as a compatibilizing agent between hydrophilic and hydrophobic components within adhesive formulations, thereby reducing the likelihood of phase separation. Despite these advantages, its hydrophilic nature has been associated with increased water sorption and permeability, which may compromise the long-term stability of the adhesive interface.^23,56^ In light of the balance between benefits and limitations and to address the drawbacks associated with one-step adhesives, some manufacturers have increasingly focused on developing HEMA-free, two-step UA systems. However, the performance of these systems may vary depending on substrate characteristics and application strategy.

Minimally invasive dentistry emphasizes the preservation of caries-affected dentin (CAD) as a clinically relevant bonding substrate.^29^ Nevertheless, most of the available evidence regarding UAs is based on sound dentin. CAD is characterized by reduced mineral content, increased porosity, and alterations in collagen structure that may adversely affect adhesive infiltration and hybrid layer formation.^24,28,36^ In addition, reduced calcium availability may limit the chemical interaction of functional monomers such as 10-methacryloyloxydecyl dihydrogen phosphate (10-MDP), potentially affecting bond durability.^37,46^ A systematic review reported that phosphoric acid pretreatment did not affect the bonding performance of ultra-mild and mild UAs to CAD.^24^ However, it remains unclear whether these findings apply to newer adhesive formulations, particularly HEMA-free and intermediately strong UAs.

Therefore, the aim of this study was to evaluate the bond strength of a HEMA-free 2-step UA to CAD before and after aging when used in both S-E and E-R protocols. The tested null hypotheses were as follows: (1) bond strength would be no different between UAs; (2) the application protocol of adhesives would not influence bond strength; (3) the substrate type would not influence bond strength; and (4) aging by thermocycling would not influence bond strength.

## Methods and Materials

### Tooth Selection and Preparation

This study utilized extracted human molars obtained for therapeutic purposes unrelated to this study, in accordance with approval from the institutional Ethics Committee (Approval No. 2025/48). Teeth exhibiting caries, restorations, or structural defects were excluded. Eligible specimens consisted exclusively of fully erupted and intact molars. For disinfection, the teeth were immersed in a 0.5% chloramine-T solution for seven days and subsequently maintained in distilled water, with storage not exceeding one month prior to specimen preparation. To expose the midcoronal dentin, each tooth was sectioned transversely using a low-speed cutting system under continuous water irrigation (Micracut 152, Metkon, Bursa, Turkey). The dentin surfaces were examined under stereomicroscopic (Stemi305, Carl Zeiss Microscopy, Göttingen, Germany) to verify the complete elimination of enamel.

The prepared specimens were randomly assigned to either the sound dentin or the artificially created CAD groups. Specimens in the sound dentin group were maintained in distilled water at 37°C to prevent dehydration. For the CAD group, all tooth surfaces, except the exposed dentin, were coated twice with an acid-resistant nail varnish to confine the demineralization process to the dentin substrate. Artificial caries-like lesions were induced using a well-established pH-cycling model,^24,51^ consisting of immersion in a demineralizing solution (2.2 mM CaCl_2_, 2.2 mM NaH_2_PO_4_, 50 mM acetic acid; pH 4.5) for 8 h, followed by immersion in a remineralizing solution (1.5 mM CaCl_2_, 0.9 mM NaH_2_PO_4_, 0.15 mM KCl; pH 7.0) for 16 h. The solutions were renewed after each cycle, and the protocol was repeated daily for 14 consecutive days at room temperature. Consequently, all dentin surfaces were gently polished with 600-grit SiC paper for 30 s under standardized light pressure to produce a uniform smear layer before the bonding procedure.^24^

To standardize the formation of CAD, specimens in the CAD group were subjected to a microhardness test before and after the induction of artificial demineralization. Dentin microhardness was determined using a microhardness device (HMV-G31DCE, Shimadzu, Kyoto, Japan) equipped with a Vickers indenter. A static load of 25 g was applied for 15 s on the dentin surface prior to the demineralization, whereas a reduced load of 10 g was applied to the demineralized dentin surface with the same dwell time. The application of different loads was required to obtain clearly defined indentations without peripheral cracking in substrates with distinct mechanical characteristics, as reported in the literature.^18,28^ These procedures were implemented to ensure consistent and standardized formation of caries-like lesions across all specimens included in the study.

### Sample Size Calculation

The sample size was determined using an a priori power analysis using G*Power software (Heinrich Heine University, Germany). The initial effect size was estimated from the shear dentin bond strength data (means and standard deviations) reported in a previous study,^20^ yielding an eta squared (η^2^) value of 0.38, which was subsequently converted to Cohen’s f (f = 0.78). However, this effect size resulted in a relatively small estimated total sample size (n = 72) for the 24 study groups. Therefore, to minimize the risk of underpowering, a more conservative effect size (f = 0.40) was adopted for the final sample size calculation.

The power analysis was performed using an F-test for one-way ANOVA with 24 groups, a significance level of α = 0.05, and 80% power. Based on these parameters, the minimum required total sample size was calculated as 168 specimens, corresponding to at least seven specimens per group. To compensate for potential specimen loss or damage during preparation and testing, the sample size was increased to 10 teeth per group. In this study, teeth were used as the statistical unit.

### Bonding Procedure

A total of 120 specimens per substrate group (sound dentin or CAD) were randomly allocated to 12 subgroups (n = 10) according to adhesive type (Clearfil S3 Bond Universal [CUB], G2-Bond Universal [G2BU], or Gluma Bond Universal [GBU]), bonding protocol (S-E or E-R), and evaluation period (24 h or aging). Adhesive systems were applied in accordance with the manufacturers’ instructions and subsequently polymerized for 10 s using a VALO LED unit (1000 mW/cm^2^, Ultradent, Utah, USA). In the E-R protocol, dentin surfaces were conditioned with 37% phosphoric acid (Panora 200, Konya, Turkey) for 15 s, rinsed with an air-water spray for 30 s, and gently dried. A polytetrafluoroethylene tube (3 mm height, 2.5 mm internal diameter) was positioned on the dentin prior to adhesive curing. Clearfil Majesty Flow resin composite (Kuraray, Tokyo, Japan) was applied in two 1.5 mm increments, and each layer was light-cured for 20 s. All procedures were conducted by a single experienced operator. The chemical composition and application procedure of the adhesives are presented in Table 1.

**Table 1:** Adhesive composition and recommended application methods

Adhesive (Manufacturer)	Chemical components	Application instructions according to manufacturers
Clearfil S3 Bond Universal (Kuraray Noritake Dental, Tokyo, Japan) Lot: 690062	10-MDP, HEMA, Bis-GMA, hydrophilic aliphatic dimethacrylate, camphorquinone, colloidal silica, accelerators, silane, ethanol, initiators, water Mild (pH = 2.3)	Apply to the dentin surface using a microbrush, actively rubbed for 10 s, gently air-dried for 5 s, and photo-cured for 10 s
G2-Bond Universal (GC Corp, Tokyo, Japan) Lot: 2406051	Primer: 10-MDP, 4-META, MDTP, water, acetone, silicon dioxide, initiators Adhesive: UDMA, dimethacrylate monomer, photoinitiator, silicon dioxide, butylated hydroxytoluene Intermediately strong (pH = 1.5)	Apply primer to dentin using a microbrush, leave undisturbed for 10 s, and gently air-dry for 5 s Apply adhesive to primed surface, gently air-thin for 5 s, and photo-cure for 10 s
Gluma Bond Universal (Kulzer, Hanau, Germany) Lot: N010067	10-MDP, 4-META, UDMA, dimethacrylate resins, acetone, silane, initiators, fillers, water Intermediately strong (pH = 1.5)	Apply to the dentin using a microbrush, actively rubbed for 20 s, air-dried until liquid does not move any longer, and photo-cured for 10 s
10-MDP: 10-Methacryloyloxydecyl dihydrogen phosphate; HEMA: Hydroxyethyl methacrylate; Bis-GMA; Bisphenol A-glycidyl methacrylate; UDMA: Urethane dimethacrylate resin; 4-META: 4-Methacryloxyethyl trimellitate anhydride; MDTP: Thiophosphate ester monomer.		

### Shear Bond Strength (SBS) Testing

Specimens were stored in distilled water at 37 °C for 24 h before the bonding test. Tubes and adhesive remnants around composite cylinders were removed with a scalpel. Each specimen was mounted on a universal testing machine (Model 3344; Instron, Canton, USA) using a specially designed jig. The dentin–composite interface was oriented parallel to the loading direction, with a precise 0.1 mm gap maintained between the crosshead and dentin surface to ensure controlled load transfer. SBS testing was performed by applying force at the interface with a sharp stainless-steel blade at a speed of 0.5 mm/min until the specimen failed. Measurements were performed either after 24 h or following 10,000 thermal cycles. Thermal cycling was conducted in a PLC-controlled apparatus (Gökçeler Makine, Sivas, Turkiye), with specimens alternately immersed in 5°C and 55°C water baths for a 30 s dwell time and 5 s transfer time, according to established protocols.^33^ The maximum load at failure was recorded in Newtons and converted to megapascals (MPa) based on the bonded area. Fracture surfaces were examined under a stereomicroscope at 40× magnification, and failure modes were categorized as adhesive, cohesive in dentin, cohesive in composite, or mixed.^5^ All procedures were performed by an operator blinded to group allocation.

### Scanning Electron Microscopy (SEM) Assessment

To assess resin tag formation and the morphology of the adhesive surface, additional specimens were prepared as previously described. Adhesive systems were applied in either S-E or E-R protocols. Composite resin was incrementally built up over the bonded dentin surfaces and polymerized for 40 s. The restored specimens were kept in distilled water at 37°C for 24 h prior to further processing. For micromorphological evaluation, the tooth structures were dissolved by immersing the specimens in 6 N hydrochloric acid until complete dentin removal was achieved. Specimens were washed with distilled water and exposed to 5% sodium hypochlorite for 10 min to eliminate residual organic components. After air-drying, specimens were sputter-coated with gold under vacuum and observed using SEM (JSM-6610, JEOL, Peabody, MA, USA) at 1500× magnification to evaluate the adhesive surface characteristics and resin tag formation.

### Degree of Conversion (DC) Assessment

The DC of the adhesives (n = 5) was determined using Fourier-transform infrared spectroscopy (FTIR; Spectrum 100, PerkinElmer, Waltham, MA, USA) equipped with an attenuated total reflectance (ATR) accessory and diamond crystal. A thin layer of each adhesive was applied directly onto the ATR crystal to ensure complete surface coverage. Spectra of the uncured materials were acquired within a wavenumber range of 650–4000 cm^–1^ using 32 scans at a resolution of 4 cm^–1^. After baseline acquisition, a gentle stream of air was applied for 5 s to facilitate solvent evaporation. To minimize oxygen inhibition, the adhesive layer was covered with a transparent Mylar strip and light-cured for 10 s, with the curing tip positioned centrally and in close proximity to the ATR crystal to standardize light exposure. Post-curing spectra were recorded under the same parameters as the uncured measurements.

ATR–FTIR spectra were processed and analyzed using OriginPro 2021 software (OriginLab, Northampton, MA, USA). The DC was determined by calculating the ratio of the absorbance of the aliphatic C=C peak (~1638 cm^–1^) to that of the aromatic C=C peak (~1607 cm^–1^) before and after polymerization, as previously described.^8^ DC values were computed using the following formula:


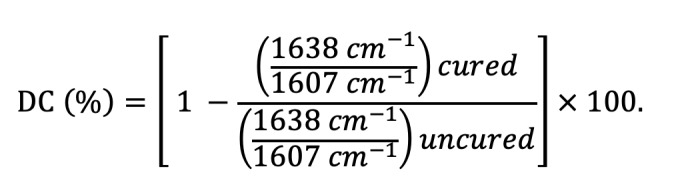


In contrast, for the GBU adhesive, which does not contain Bis-GMA, the carbonyl (C=O) absorption band at 1716 cm^–1^ was used as the internal reference peak, since this material lacks an aromatic benzene ring structure. Accordingly, DC was calculated using the following equation^61^:


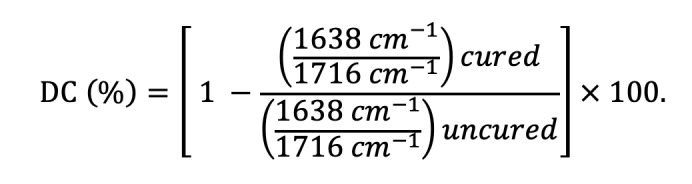


### Statistical Analysis

The distributional assumptions were first evaluated using the Kolmogorov–Smirnov test for normality and Levene’s test for homogeneity of variances. After confirming that these assumptions were satisfied, microhardness values were compared using a paired t-test. SBS data were analyzed using a four-way ANOVA, followed by Tukey’s post hoc test (*p* < 0.05). The reliability of adhesion and the probability of failure were further assessed through Weibull analysis. The Weibull shape (m) and scale (σθ) parameters were calculated using the maximum likelihood estimation method. The shape parameter indicates the reliability of the material, whereas the scale parameter corresponds to the bond strength at a 63.2% probability of failure. Kolmogorov–Smirnov test revealed that the DC findings were not normally distributed (*p* = 0.007). DC values were assessed using the Kruskal–Wallis test with Bonferroni adjustment (*p* < 0.05).

## RESULTS

### Microhardness Test Analysis

Sound dentin exhibited significantly greater microhardness compared with demineralized dentin, confirming substantial mineral loss following the demineralization protocol (*p* < 0.001; Table 2).

**Table 2:** Microhardness values (± SD) for the different substrates

Substrate	95% Confidence interval		Mean	t	*p*
	Lower bound	Upper bound			
Sound dentin	45.53	51.74	48.64 ± 5.51^A^	24.21	< 0.001
Caries-affected dentin	10.10	11.06	10.58 ± 1.16^B^		
Different letters indicate significant differences.					

### SBS Analysis

The mean SBS values (± SD) are summarized in Table 3. The adhesive system and bonding protocol had a significant effect on SBS (*p* < 0.001). In contrast, neither dentin substrate (*p* = 0.056) nor thermal cycling (*p* = 0.095) significantly influenced bond strength. A significant interaction was observed only between adhesive type and substrate condition (*p* = 0.009), whereas no other factor interactions reached statistical significance (*p* > 0.05).

**Table 3:** Mean shear bond strengths (± SD) for adhesives on different substrates

Adhesives	Application mode	Sound dentin		Caries-affected dentin	
		24 h	Ageing	24 h	Aging
GBU	S-E	11.87 ± 3.23^Aab^	9.95 ± 3.93^Aab^	12.27 ± 4.99^Aa^	12.68 ± 2.17^Ab^
	E-R	8.10±5.16^Aa^	7.85 ± 4.82^Aa^	10.41 ± 2.54^Aa^	5.70 ± 5.19^Aa^
CUB	S-E	7.64 ± 3.41^Aa^	8.49 ± 3.33^Aa^	8.55 ± 1.88^Aa^	5.95 ± 3.45^Aa^
	E-R	7.58 ± 2.40^Aa^	5.17 ± 3.07^Aa^	6.25 ± 4.56^Aa^	5.39 ± 4.57^Aa^
G2BU	S-E	15.34± 3.16^Ab^	15.99 ± 4.13^Ab^	11.20 ± 6.04^Aa^	14.22 ± 4.82^Ab^
	E-R	11.85 ± 4.38^Aab^	10.85 ± 6.45^Aab^	8.80 ± 4.73^Aa^	6.75 ± 4.20^Aa^
Different uppercase letters indicate significant differences horizontally, and different lowercase letters indicate significant differences vertically (*p* < 0.05).					

**Fig 1:**
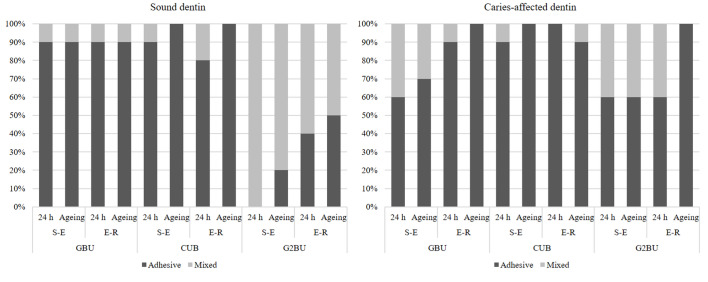
Stereomicroscopy failure analysis presenting the failure-mode distribution of adhesives on different substrates in S-E or E-R protocols.

At both 24 h and after aging, G2BU in the S-E protocol exhibited the highest SBS values on sound dentin, showing a significant difference compared with CUB (*p* < 0.001). At 24 h, no significant differences were found among the adhesives on CAD, irrespective of the bonding protocol. Following aging, CUB in the S-E protocol demonstrated significantly lower SBS to CAD than G2BU (*p* = 0.004) and GBU (*p* = 0.038), whereas no significant difference was detected between G2BU and GBU (*p* = 0.77). After aging, G2BU and GBU achieved significantly higher SBS values in the S-E protocol compared with the E-R protocol on CAD, while CUB showed no significant difference between bonding protocols. In the E-R protocol, SBS values were statistically comparable among all adhesives, regardless of substrate type or evaluation period. Failure mode analysis revealed predominantly adhesive failures for CUB and GBU on sound dentin, regardless of bonding protocol and testing time. In contrast, G2BU mainly exhibited mixed failures (adhesive and cohesive in the composite). On CAD, adhesive failure was the predominant mode for all adhesive systems (Fig 1).

**Fig 2:**
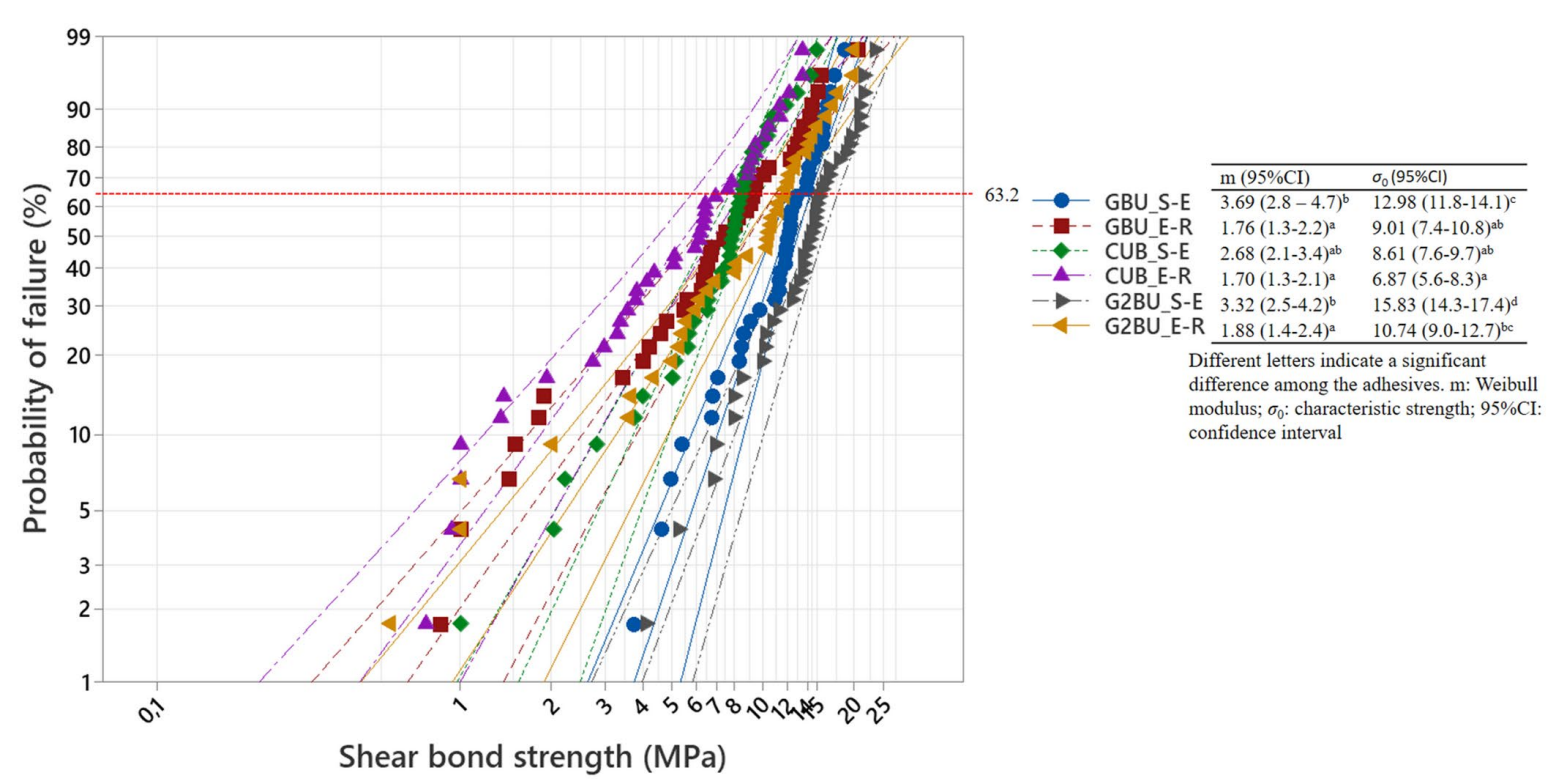
Weibull analysis of adhesive failure probability, regardless of substrate type and testing time (n = 40). Relationship between the Weibull parameters, where m represents material reliability and σθ denotes the bond strength at a 63.2% probability of failure; statistical differences were considered significant when the 95% CIs did not overlap.

Figure 2 illustrates the Weibull reliability analysis and corresponding probability of failure for the tested adhesives (n = 40), irrespective of substrate type and evaluation period. Overall, adhesives applied in the S-E protocol demonstrated significantly greater reliability than those applied in the E-R protocol, except for CUB, which showed no statistically significant difference between bonding protocols. Within each bonding protocol (S-E or E-R), the adhesives exhibited comparable reliability. G2BU in the S-E protocol showed the lowest probability of failure among the adhesives, with failures occurring at higher stress levels during mechanical loading. In addition, both G2BU and GBU demonstrated a significantly lower probability of failure under the S-E protocol than under the E-R protocol. In contrast, CUB exhibited no significant difference in failure probability between the two bonding protocols.

### SEM Analysis

**Fig 3:**
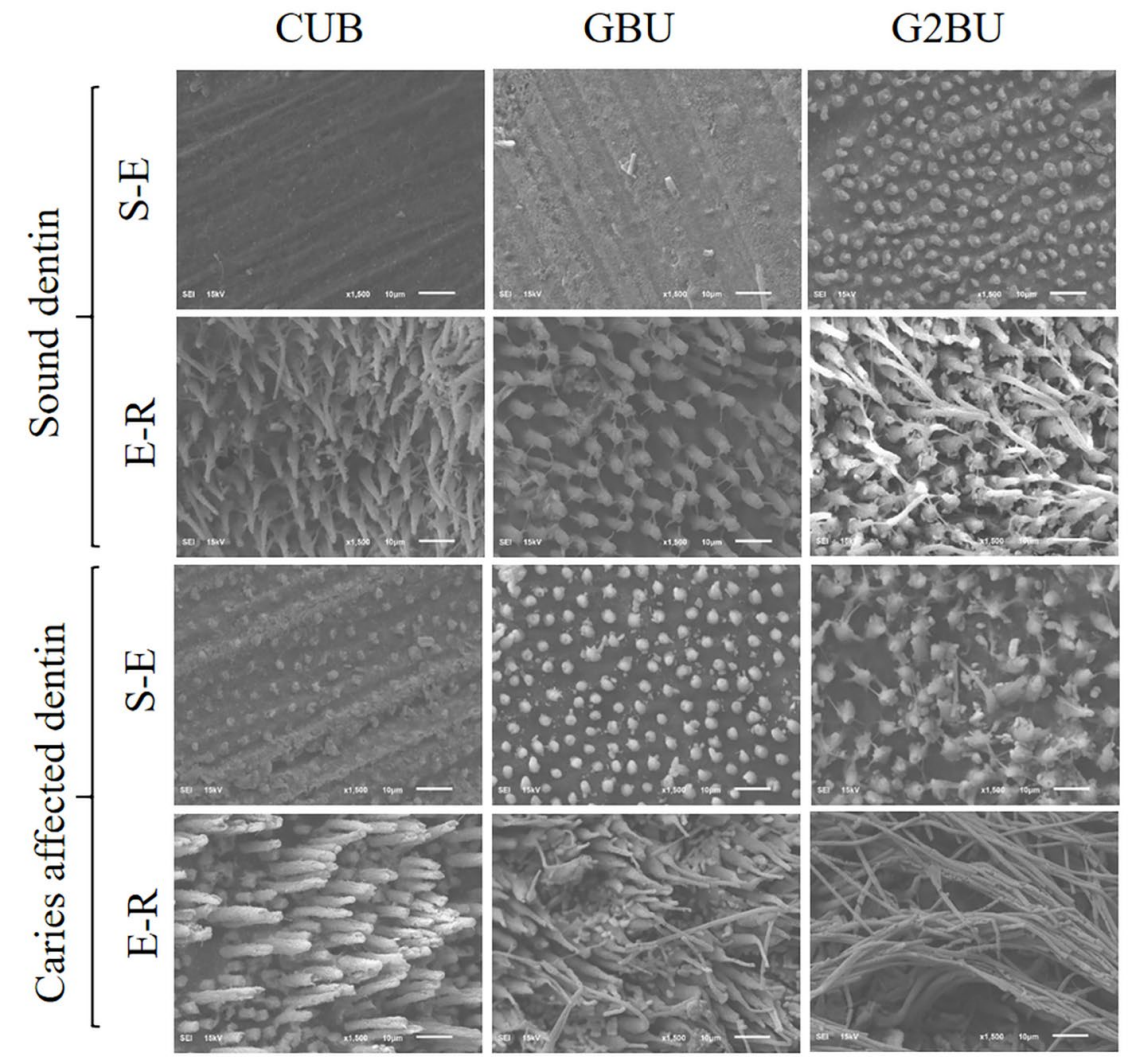
Representative SEM photomicrographs of adhesive surfaces on different dentin surfaces. S-E: self-tech; E-R: etch-and-rinse; CUB: Clearfil S3 Bond Universal; GBU: Gluma Bond Universal; G2BU: G2-Bond Universal.

Figure 3 shows SEM images of the adhesive layer formed on dentin surfaces using adhesives applied in S-E and E-R protocols. Morphological differences were observed between the adhesive layers formed with the two application protocols. In sound dentin, short resin tags within the dentinal tubules were observed only in G2BU in the S-E protocol, whereas no resin tag formation was detected in the other adhesives. In CAD, short resin tags were observed for all adhesives, which were more pronounced for G2BU and GBU in the S-E protocol. After phosphoric acid etching, resin infiltration into the intra- and inter-tubular dentin was observed, and long resin tags were detected in all adhesive groups. CAD revealed a higher number of resin tags compared to sound dentin. The highest number of resin tags may be seen in G2BU, regardless of dentin substrate.

### DC Analysis

**Fig 4:**
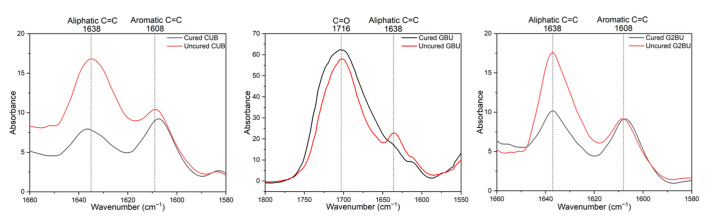
ATR-FTIR spectra for universal adhesives.

The DC values (± SD) are presented in Table 4. Significant differences in DC were found among the adhesive systems (*p* = 0.014). CUB demonstrated a significantly higher DC compared with both GBU and G2BU, whereas no significant difference was found between GBU and G2BU. Representative ATR–FTIR spectra obtained before and after light-curing are presented in Figure 4.

**Table 4:** Comparison of conversion degree values (± SD) of the adhesives

Adhesive	Min-max	Mean	Median	Test statistics	*p*
CUB	46.68–66.46	61.31 ± 8.25	64.54^A^	8.571	0.014
G2BU	41.79–43.71	42.90 ± 0.88	43.30^B^		
GBU	30.22–45.03	40.27 ± 5.82	42.34^B^		
Different letters indicate significant differences (*p* < 0.05).					

## DISCUSSION

This study evaluated the bonding potential to CAD of UAs with or without HEMA. Statistical analysis revealed that bonding strength significantly differed among UAs tested, regardless of testing time and substrate. Thus, the first null hypothesis that bond strength would be no different between UAs was rejected.

The two-step G2BU system (primer and adhesive) showed the highest bonding performance in the S-E protocol, but statistically similar performance to GBU on sound dentin or CAD. Given the inherent variability of bond strength data, as observed in this study,^39^ Weibull analysis was employed to provide a more comprehensive assessment of adhesive reliability and failure probability.^22^ While all adhesives exhibited comparable reliability within each bonding protocol (S-E or E-R), G2BU in the S-E protocol demonstrated a notably lower probability of failure, with failures occurring at higher stress levels during mechanical loading. SEM images also revealed that G2BU showed better penetration into the dentin surface and short resin tags. The acidic nature of the G2BU primer may be associated with improved smear layer modification and primer monomer infiltration into dentin. Effective dentin bonding depends on surface wettability, micromechanical retention, and stable chemical interactions. In S-E adhesive systems, bonding mainly occurs through chemical interactions between hydroxyapatite and functional monomers. In two-step S-E adhesives, hydrophilic components improve monomer penetration through the smear layer and partially demineralized dentin, leading to stronger chemical adhesion with mineralized structures. In contrast, single-step S-E adhesive agents combine etching, priming, and bonding in one bottle, which may reduce the overall effectiveness of the bonding process.^54,56^ Although UAs tested contain 10-MDP, the success of this interaction depends on sufficient monomer infiltration and the formation of a uniform and durable hybrid layer.^63^

In this study, GBU presented higher dentin bonding performance than CUB when used in the S-E protocol (Fig 2). GBU is an intermediately strong S-E adhesive (pH ≈ 1.5), whereas CUB is a mild S-E adhesive (pH ≈ 2.5). The lower pH of GBU promotes greater dissolution of the smear layer and superficial demineralization of dentin, facilitating deeper penetration of the monomer. SEM observations, especially in CAD, confirmed more pronounced demineralization and more distinct resin tag formation with GBU. These features contribute to a higher micromechanical interlocking, which is associated with improved bond strength.^56,64^ In addition, GBU contains 4-META monomer, which can chemically interact with residual hydroxyapatite and enhance interfacial stability. In contrast, CUB contains HEMA, enhancing initial wettability but potentially increasing adhesive permeability.^1^ Furthermore, the absence of HEMA has been reported to promote the formation of a more stable and well-organized 10-MDP nano-layering at the bonding interface, which contributes to enhanced bonding durability, particularly in the S-E protocol.^49,56,62^ Consequently, the combined effects of stronger acidity and favorable monomer composition may explain the higher bonding performance observed with GBU.

After aging, GBU and G2BU exhibited significantly lower bonding performance in the E-R protocol compared with the S-E protocol on CAD, whereas CUB showed comparable performance in both protocols. Thus, the second null hypothesis that the application protocol of adhesives would not influence bond strength was rejected. GBU and G2BU are acetone-based and HEMA-free adhesives. Previous studies have demonstrated that solvent type can play a critical role in bonding effectiveness to etched dentin.^25,41^ In acetone-based systems, strict wet-bonding is required to prevent collapse of the demineralized collagen network due to acetone’s strong water-chasing effect.^38^ The reduced performance observed may therefore be attributed to insufficient resin monomer infiltration into etched dentin, largely associated with acetone’s high volatility and limited ability to maintain collagen fibril expansion.^23^ Moreover, the absence of HEMA may further compromise bonding to moist, etched dentin by reducing monomer compatibility, thereby increasing the risk of phase separation, water entrapment, and microvoid formation during solvent evaporation. These factors collectively impair hybrid layer integrity and reduce micromechanical retention.^23,56^ In contrast, CUB is ethanol-based and contains HEMA. Ethanol facilitates gradual water displacement and, due to its water miscibility, helps preserve collagen fibril expansion, promoting more uniform resin infiltration within the demineralized dentin matrix.^57^

In the E-R protocol, no significant differences were detected among the adhesives, irrespective of the substrate or testing period. Within this approach, dentin bonding effectiveness is predominantly governed by micromechanical retention mechanisms, particularly the formation of a well-defined hybrid layer and adequate resin tag penetration.^11,23^ Following phosphoric acid etching, adhesive monomers diffuse into the demineralized, microretentive dentin matrix, resulting in the creation of a hybrid layer several micrometers thick.^57^ Although secondary interactions such as van der Waals forces and hydrogen bonding may occur, their contribution to dentin adhesion is limited, and the formation of stable chemical bonds with the exposed collagen matrix remains unlikely.^11^ On the other hand, all adhesives in the E-R protocol demonstrated lower reliability than those in the S-E protocol, particularly GBU and G2BU. S-E adhesives simultaneously demineralize dentin and infiltrate resin monomers, thereby minimizing the risk of collagen collapse and incomplete resin penetration. Moreover, the partial preservation of hydroxyapatite around collagen fibrils in the S-E protocol enables functional monomers, such as 4-META and 10-MDP, to establish chemical interactions with residual hydroxyapatite, resulting in a more reliable and stable adhesive interface.^57^ In contrast, in the E-R protocol, excessive demineralization followed by insufficient resin infiltration, particularly in CAD, may compromise bonding effectiveness and restrict bond strength.^5^ Also, a previous study reported that MMP enzymes are released in greater amounts in the E-R protocol than in the S-E protocol.^10^ This enzymatic activity breaks down collagen fibrils, compromising the integrity of the hybrid layer and reducing the mechanical strength of the dentin–resin interface.

In the present study, the dentin substrate did not affect the bond strength of the tested adhesives. Thus, the third null hypothesis that the type of substrate would not influence bond strength was accepted. Although limited data are available in the literature, several studies have reported comparable or clinically acceptable bond strength values in CAD.^32,47^ Conflicting results have been reported regarding the bond strength of UAs to sound dentin versus CAD. While some studies have demonstrated significantly higher bond strength to sound dentin,^27,28,30^ others have found no significant difference between the two substrates.^16,42,59^ These differences may be attributed to methodological variations, including differences in the type of adhesive used, smear layer preparation, and the characteristics of the carious dentin. To the best of our knowledge, no previous studies have tested UAs employed in the present study on CAD. Consequently, direct comparisons of the adhesives tested here with previous literature are limited. CAD is characterized by reduced mineral content and increased organic matrix, which may compromise the ability of functional monomers such as 10-MDP to establish stable chemical bonds with hydroxyapatite due to the decreased availability of calcium ions.^24^ Nevertheless, SEM analysis revealed the formation of more pronounced and numerous resin tags in the S-E and E-R protocols in CAD. This enhanced micromechanical interlocking may compensate for the reduced chemical interaction, thereby supporting the observed bonding performance and explaining the comparable bonding strength values between sound dentin and CAD.

In the present study, thermocycling did not affect the bond strength of the tested adhesives, irrespective of the dentin substrate. Thus, the fourth null hypothesis that aging by thermocycling would not influence bond strength was accepted. Previous studies have reported conflicting findings regarding the effect of thermocycling on bond strength,^7,9,26,53^ which have been mainly attributed to differences in adhesive mixture, thermocycling protocols, and specimen design. The use of large-diameter composite cylinders bonded to flat dentin surfaces may limit thermal stresses at the adhesive interface and reduce shrinkage- and temperature-induced stresses due to the low C-factor.^26,58^ Accordingly, thermocycling applied to flat surfaces has been reported to have minimal influence on bond strength, whereas the same aging protocols applied to restored cavities result in a significant reduction in bonding performance.^31^ In contrast, specimens with short diffusion pathways have shown increased susceptibility to interfacial degradation after thermocycling, leading to reduced bonding strength.^60^

The DC is a crucial parameter that influences the polymerization efficiency of dental adhesives and, consequently, their bond strength.^45,52^ However, conflicting results have been reported regarding the relationship between DC and the bonding performance of adhesives.^4,12,43,52^ Thicy et al^52^ reported a positive correlation between bond strength and DC, which became more pronounced after aging. In contrast, other studies have found no significant correlation between bond strength and DC.^4,43^ Complete monomer conversion is never achieved in adhesive systems, and DC values generally range from 35% to 77%, which is lower than that observed for composite resins.^14,15,48^ In the current study, CUB indicated a significantly higher DC than GBU and G2BU, which exhibited similar DC values. The type of solvent in the adhesive plays a critical role in polymerization efficiency. Acetone-based, HEMA-free adhesive systems can lead to water accumulation within the adhesive layer and the formation of voids, known as ‘water trees,’ which may inhibit monomer polymerization.^50,55^ The lower DC observed for the HEMA-free adhesives in the current study may therefore be attributed to these solvent-related effects. However, DC was measured immediately after polymerization, and previous studies have shown that DC may increase over time, indicating that the timing of measurement can influence the results.^2,43^

In the present study, shear bond strength testing was employed to evaluate adhesion to dentin. Although microtensile testing is often considered the gold standard for assessing bond strength, shear testing offers several practical and methodological advantages. The stresses occurring at the tooth–restoration interface in the oral environment are complex, but predominantly manifest as tensile and shear forces; therefore, shear tests are better suited to simulate these conditions.^21,44^ Comparative studies have demonstrated that shear bond tests can provide higher and clinically relevant bond strength values while maintaining reproducibility.^6,13^ In addition, shear testing is less technique-sensitive, easier to standardize, and allows the evaluation of a larger number of specimens in a shorter time frame, whereas microtensile testing requires precise specimen preparation, specialized equipment, and greater operator skill, which may limit its feasibility in routine laboratory studies.^35,40^

This study has several limitations. First, it was performed under *in vitro* conditions, and the findings may not fully replicate clinical variables such as oral humidity, pH fluctuations, mechanical stresses, and biological interactions, all of which can influence adhesive performance.^17^ Although the pH-cycling model provides controlled demineralization, it may not fully reflect the biofilm-mediated natural caries process, and this may limit the clinical generalizability of the results. Second, the study involved 10,000 thermal cycles, corresponding to approximately one year of clinical period, which may be considered a relatively short period for predicting long-term outcomes. Additionally, factors such as cavity configuration (C-factor) and occlusal loading were not considered. Therefore, long-term clinical studies are necessary to validate these results and to better understand the durability and performance of these adhesives under real-life conditions.

## CONCLUSION

In the S-E protocol, G2BU demonstrated the highest bonding performance, followed by GBU, regardless of testing time and dentin substrate. The effect of phosphoric acid application on bonding performance was dependent on the adhesive system. Substrate type and thermocycling did not affect the bonding performance of the adhesives tested. However, these findings should be interpreted with caution due to the *in vitro* nature of the study.

### Acknowledgments

#### Funding statement (open access)

This research has been supported by the Recep Tayyip Erdoğan University Development Foundation (grant number: 02026003030214).

This study was funded by the Research Fund of University (project no. TDH-2023-1493).

#### Clinical relevance

G2-Bond Universal exhibited the lowest probability of failure in the self-etch protocol, regardless of substrate type. Universal adhesives demonstrated greater reliability in the self-etch protocol than in the etch-and-rinse protocol.
